# Antibody response after first and second BNT162b2 vaccination to predict the need for subsequent injections in nursing home residents

**DOI:** 10.1038/s41598-022-18041-x

**Published:** 2022-08-12

**Authors:** Edouard Tuaillon, Amandine Pisoni, Nicolas Veyrenche, Sophia Rafasse, Clémence Niel, Nathalie Gros, Delphine Muriaux, Marie-Christine Picot, Safa Aouinti, Philippe Van de Perre, Jean Bousquet, Hubert Blain

**Affiliations:** 1 Pathogenesis and Control of Chronic and Emerging Infections, University Montpellier, INSERM, Établissement Français du Sang, Antilles University, CHU Montpellier, Montpellier, France; 2 CEMIPAI, University of Montpellier, UAR3725 CNRS, Montpellier, France; 3 Clinical Research and Epidemiology Unit, University Hospital, Montpellier, France; 4 Department of Dermatology and Allergy, Charité, Univeersitätsmedizin Berlin, Humboldt-Universität zu Berlin, and Berlin Institute of Health, Comprehensive Allergy Center, Berlin, Germany; 5 University Hospital, Montpellier, France; 6 Department of Geriatrics, Montpellier University Hospital, Montpellier University, Montpellier, France

**Keywords:** Infectious-disease diagnostics, Infectious diseases, Vaccines, SARS-CoV-2, Viral host response

## Abstract

We explored antibody response after first and second BNT162b2 vaccinations, to predict the need for subsequent injections in nursing home (NH) residents. 369 NH residents were tested for IgG against SARS-CoV-2 Receptor-Binding Domain (RBD-IgG) and nucleoprotein-IgG (SARS-CoV-2 IgG II Quant and SARS-CoV-2 IgG Alinity assays, Abbott Diagnostics). In NH residents with prior SARS-CoV-2 infection, the first dose elicited high RBD-IgG levels (≥ 4160 AU/mL) in 99/129 cases (76.9%), with no additional antibody gain after the second dose in 74 cases (74.7%). However, a low RBD-IgG level (< 1050 AU/mL) was observed in 28 (21.7%) residents. The persistence of nucleoprotein-IgG and a longer interval between infection and the first dose were associated with a higher RBD-IgG response (p < 0.0001 and p = 0.0013, respectively). RBD-IgG below 50 AU/mL after the first dose predicted failure to reach the antibody concentration associated with a neutralizing effect after the second dose (≥ 1050 AU/mL). The BNT162b2 vaccine elicited a strong humoral response after the first dose in a majority of NH residents with prior SARS-CoV-2 infection. However, about one quarter of these residents require a second injection. Consideration should be given to immunological monitoring in NH residents to optimize the vaccine response in this vulnerable population.

## Introduction

Nursing home (NH) residents accounted for a large proportion of the excess deaths during the first and second waves of the SARS-CoV-2 epidemic in France^1^. Hence, NH residents were among those groups prioritized in the vaccination campaign launched in January 2021 by the French authorities. In March 2021, almost all of around one million NH residents had received a first vaccine dose, with three quarters having received two doses. This strategy led to a sharp drop in excess deaths in NHs. However, COVID-19 outbreaks continued to occur in NHs after mRNA vaccination^2–4^. Studies in NHs reported a vaccine efficacy against SARS-CoV-2 infection of around 75% after two doses^4^.

A high degree of immunity is necessary to protect residents against severe COVID-19, and to keep the spread of infection at a low level, given that communal spaces are shared in NHs. However, heterogeneity in the immune response to vaccination is observed in the elderly^5^. SARS-CoV-2 infection history is the strongest predictor of anti-spike antibody response^6^. A second vaccine dose does not increase antibody levels in pre-immunized health care workers^7^. It remains unclear whether or not a second dose administered shortly after the first one is needed to maximize vaccine effectiveness in NH residents previously exposed to SARS-CoV-2^8^. It is also unclear whether the second dose was sufficiently effective to allow a 6-month delay before administering the third dose to SARS-CoV-2 naïve residents. The first dose of mRNA vaccine induces a strong RBD-IgG response in individuals with prior COVID-19^9,10^. Given that older adults may have a limited vaccine response, NH residents in France received two vaccine doses regardless of any prior SARS-CoV-2 infection. However, most NH residents with evidence of previous infection showed a strong humoral response after a single BNT162b2 dose, suggesting that recommendations for immunocompetent individuals could also be applied to this population^11^. Conversely, a lack of or low antibody vaccine response was observed in NH residents without prior SARS-CoV-2 infection^11,12^.

Authorization for the use of the two mRNA vaccines encoding SARS-CoV-2-spike was based on the results of phase 3 clinical trials^13,14^. While these vaccines have proven high levels of efficacy in preventing severe forms of illness in the general population, it has yet to be evaluated whether immune responses elicited by SARS-CoV-2 mRNA vaccines are homogenously robust in the elderly^15,16^.

It remains unclear if the humoral response after one BNT162b2 dose is predictive of the response subsequent to two doses in older adults, and how natural immunization and the interval since infection may modulate vaccine response. A better understanding of the consequences of SARS-CoV-2 infection history and of antibody response to the first vaccine dose is needed in order to adjust vaccine policies for this vulnerable population.

In this study, in a large cohort of NH residents, we assessed the value of RBD-IgG levels after the first BNT162b2 dose to predict: (i) the lack of additional benefits from a second dose as to the generation of antibodies, (ii) a significant antibody response after the second dose, and (iii) a prior SARS-CoV-2 infection. We also assessed the impact on vaccine response of natural serological status against SARS-CoV-2 nucleocapsid, and of the interval since infection.

## Methods

### Design of the study

All experiments were performed in accordance with relevant guidelines and regulations. Our study is part of the CONsort-19 cohort^10,17–19^ a longitudinal follow-up of NH residents performed during the COVID-19 pandemic. In this study, we monitored IgG against Receptor-Binding Domain (RBD-IgG) 3 weeks after the first BNT162b2 dose, and 6 weeks after the second dose, considered as a period in which the antibody decay rate response is stabilized^20^. Results in residents without prior COVID-19 vs. residents with prior COVID-19 were compared. Two periods were considered for COVID-19 recovery—the past 9–12 months (older infections), and the past 3–7 months (newer infections)—corresponding to the first and second waves of the epidemic in our region. RBD-IgG levels before vaccination and their relationship to vaccine response were analyzed in a subgroup of residents. We also assessed IgG levels against N-protein 6 weeks after a second BNT162b2 dose in residents infected in the previous 9–12 months vs. the previous 3–7 months, and we evaluated the impact of N-IgG status on RBD-IgG vaccine response.

### Setting and participants

Invited to participate in the study were residents from nine NHs which had suffered COVID-19 outbreaks in 2020, and who were already included in the CONsort-19 cohort^17–19^. Residents were included after having signed an informed consent. Participants received the first dose of the BNT162b2 vaccine between January and March 2021, and received the second dose 3 weeks later. Blood collections were performed 3 weeks after the first dose and 6 weeks after the second dose. In accordance with the regional Health Agency (Occitanie, South of France) and the European Geriatric Medicine Society guidance^17^, as soon as a case of COVID-19 was diagnosed in a NH, all residents and healthcare professionals were repeatedly tested using PCR on nasopharyngeal swabs until no new cases were diagnosed. Residents were grouped by date of PCR positivity (wave 1 and 2). Residents tested negative by PCR but positive for SARS-CoV-2 nucleocapsid IgG the day before the second dose were also considered as having a prior infection without ruling on the occurrence of the SARS-CoV-2 infection during the first or second wave. The study was approved by the Montpellier University Hospital institutional review board (IRB-MTP_2020_06_202000534 and IRB-MTP _2021_04_202000534).

### Laboratory methods

S-protein IgG against the SARS-CoV-2 Receptor-Binding Domain (RBD) of the S1 subunit was quantified using the SARS-CoV-2 IgG II Quant assay, a chemiluminescent microparticle test using the Alinity i automatic analyzer (Abbott Diagnostics). The Abbott IgG II method provides results linearity over a wide concentration range with good agreement with external calibration materials^21^. Results were expressed as arbitrary units per mL (AU/mL; positive threshold: 50 AU/mL; upper limit: 40,000 AU/mL). RBD-IgG levels in AU/mL can be converted in Binding Antibody Units (BAU) following manufacturers’ recommendations (AU/mL × 0.142). Serum dilutions were performed to quantify RBD-IgG when the results surpassed the higher limit of quantification of the assay. According to previous studies and manufacturer’s instructions, a first threshold ≥ 1050 AU/mL was considered as a significant response^22^, and a second threshold ≥ 4160 AU/mL as a level indicating a high neutralizing effect^23^. We performed microneutralization assays to confirm these thresholds. A panel of 15 sera was tested against SARS-CoV-2 and a B.1.1.7 variant (Alpha VOC). RBD-IgG concentration was significantly correlated with neutralizing antibody titers (Spearman correlation coefficients of R^2^ = 0.89 for wild type strain and 0.90 for B.1.1.7 (p < 0.0001) (Supplemental Fig. 1). As regards RBD-IgG levels, neutralizing activities were (i) high when over 4160 AU/mL (neutralizing antibody titer of 640 log IC50 (IQR: 640–1960) for wild type SARS-CoV-2, 640 log IC50 (IQR: 640–1920) for B.1.1.7 strain), (ii) absent or moderate when between 1050 and 4160 AU/mL, and (iii) absent when below 1050 AU/mL.

A rise in RBD-IgG concentration of 50% (1.5 fold) was considered as significant. Nucleoprotein-IgG was tested on specimens collected during the first sampling using the SARS-CoV-2 IgG assay (Abbott Diagnostics). Results were expressed as signal to cut-off ratio (S/CO). A nucleoprotein-IgG ratio of over 0.5 was retained as positive for a prior SARS-CoV-2 infection.

### Statistical analysis

Qualitative variables were described with frequency and proportions for each category. The description of quantitative variables was performed using mean with standard deviation or median with interquartile range, depending on the normality of the results. To describe the distribution of RBD-IgG response, RBD-IgG levels were grouped in the following categories: < 50 (negative), 50–149, 150–299, 300–599, 600–1049, 1050–4159, 4160–19,999, 20,000–39,999, > 40,000 AU/mL. For categorical variables, comparisons of percentage were made with Chi-square test or Fisher’s exact test if Chi-square was not a valid test. Paired *t* tests were used for a comparison of changes in IgG-RBD levels for the same subject. Two multivariate logistic regressions were used to analyze the determinants of high RBD-IgG response (> 4160 AU/mL) after the first and second doses respectively, adjusted according to the resident’s characteristics (age, sex, first or second wave prior COVID-19 and N-IgG). The receiver operating characteristic curves (ROCs) were used to identify RBD-IgG levels after the first vaccine dose, as predictive of: (i) a lack of additional beneficial effect on RBD-IgG level after the second dose; (ii) a poor or a robust humoral response after the second dose; and (iii) a previous SARS-CoV-2 infection. Wilcoxon–Mann–Whitney 2-sided tests were used for quantitative variables. A paired *t* test or Signed-ranked Wilcoxon test was used for comparison of IgG-RBD levels in the same participant. Spearman’s non-parametric test was used to assess correlation between IgG-RBD levels after the first and second doses, and after SARS-CoV-2 infection and the first dose. The statistical significance threshold was set at 5%. Statistical analyses and graphs were performed using GraphPad Prism 9.1.1 (GraphPad Software, Inc., San Diego, CA).

## Results

### Demographic characteristics

The characteristics of the 396 residents included in the study are presented in Table 1. One hundred and twenty-nine residents had a prior infection (32.6%). Among the PCR-positive residents, 51 had been infected during the first COVID-19 wave (March–June 2020) and 68 during the second wave (October–December 2020). Among the 129 residents with prior SARS-CoV-2 infection, 89 (69.0%) tested positive for both SARS-CoV-2 RNA and nucleoprotein-IgG, 30 (23.2%) tested positive for SARS-CoV-2 RNA only and 10 (7.8%) tested positive for nucleoprotein IgG only.

**Table 1:** Characteristics of the participants.

	Total	NH 1	NH 2	NH 3	NH 4	NH 5	NH 6	NH 7	NH 8	NH 9
Number of participants	396	23	63	36	72	38	36	47	42	39
Age mean (SD), years	86.0 (10.2)	80.7 (12.8)	84.3 (11.0)	81.2 (14.6)	89.5 (8.5)	86.6 (10.7)	86 (8.1)	85.9 (8.5)	88.5 (7.4)	86.7 (8.2)
Female/male	276/120	18/5	44/19	23/13	54/18	29/9	26/10	31/16	27/15	24/15
Prior SARS-CoV-2 (%)	129 (32.6)	3 (13)	6 (9.5)	18 (50.0)	35 (48.6)	8 (21.0)	14 (38.9)	14 (29.8)	9 (21.4)	22 (56.4)
Nucleoprotein IgG+ (%)	99* (76.7)	2 (66.7)	4 (66.7)	13 (72.2)	27 (77.1)	5 (62.5)	8 (57.1)	13 (92.8)	7 (77.8)	20 (90.9)
Covid-19 waves 1/2**	51/68	1/2	5/0	17/0	0/33	7/0	0/14	12/0	9/0	0/19

*89 tested positive for SARS-CoV-2 RNA.

**COVID-19 proved by PCR.

### The RBD-IgG level after the first dose predicted the level after the second dose

**Figure 1:**
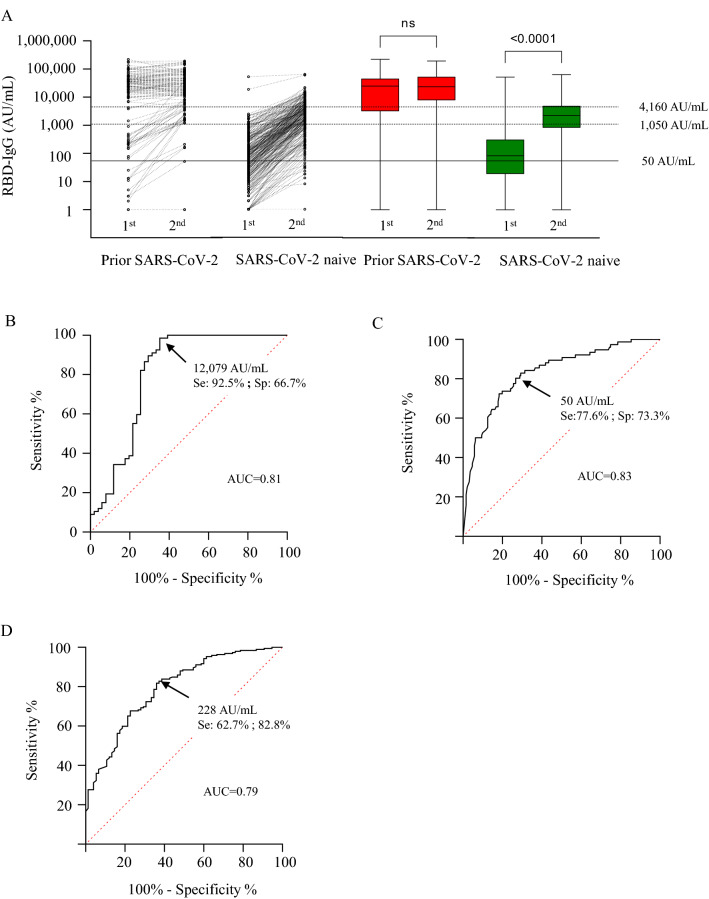
RBD-IgG vaccine response after first and second doses. (**A**) Individual changes in RBD-IgG levels after first and second doses in NH residents with and without prior SARS-CoV-2 infection. RBD-IgG between first and second doses in NH residents with (red) and without (green) prior SARS-CoV-2 infection. (**B**) ROCs to evaluate RBD-IgG levels after the first dose, predictive of the lack of additional gain in RBD-IgG following the second dose in NH residents with prior SARS-CoV-2 infection; for a RBD-IgG level > 12,079 AU/mL: positive predictive value (PPV): 76.7%, negative predictive value (NPV): 54.5%, sensitivity: 92.5%; specificity: 66.7%. (**C**) ROC evaluating RBD-IgG levels after the first dose, predictive of the failure to reach a significant RBD-IgG level after the second dose (< 1050 AU/mL) in NH residents without prior SARS-CoV-2 infection; RBD-IgG < 50 AU/mL: PPV: 53.5%; NPV: 89.2%; sensitivity: 77.6%; specificity: 73.3%. (**D**) ROC to evaluate the RBD-IgG level after the first dose, predictive of a high RBD-IgG level following the second dose (≥ 4160 AU/mL) in NH residents without prior SARS-CoV-2 infection; RBD-IgG > 228 AU/mL: PPV: 58.8%; NPV: 85.0%; sensitivity: 82.8%; specificity: 62.7%.

In residents with prior SARS-CoV-2 infection, the first dose induced a high RBD-IgG level in most individuals (median; IQR: 24,617; 3909–43,692 AU/mL). RBD-IgG level was not significantly different after the second dose (23,341; 8112–50,341 AU/mL) (Fig. 1A). Twenty-eight residents (21.7%) had levels of below 1050 AU/mL after the first dose. Six participants (4.6%) failed to produce RBD-IgG levels of over 1050 AU/mL after the second dose, and two (1.5%) remained seronegative for RBD-IgG (< 50 AU/mL). A high RBD-IgG level after the first dose predicted the lack of RBD-IgG gain after the second dose (Fig. 1A).

In residents without prior SARS-CoV-2 infection, the first vaccine dose generally elicited a low RBD-IgG response (median; IQR: 83; 20–302 AU/mL). The second dose increased RBD-IgG levels significantly, but the antibody vaccine response remained lower by comparison with residents with prior SARS-CoV-2 infection (2236; 842–4861 AU/mL, p < 0.0001). Antibody response remained low after the second dose for a quarter of the residents without prior SARS-CoV-2 infection. Ten (3.7%) residents tested negative for RBD-IgG, and 66 (24.7%) had a low RBD-IgG response (50–1050 AU/mL) (Fig. 1A).

In NH residents with prior SARS-CoV-2 infection, a high RBD-IgG level after the first dose predicted a lack of additional gain following the second dose (Fig. 1B). In NH residents without prior SARS-CoV-2 infection, an absence of RBG-IgG after the first dose predicted the failure to reach a significant antibody concentration (≥ 1050 AU/mL) after the second dose (PPV: 53.5%, NPV: 89.2%, sensitivity: 77.6%, specificity: 73.3%) (Fig. 1C). Conversely, an RBG-IgG level of over 228 AU/mL after the first dose predicted the capacity to reach a high RBD-IgG concentration (≥ 4160 AU/mL) after the second dose (PPV: 58.7%, NPV: 87.0%, sensitivity: 62.7%, specificity: 82.8%) (Fig. 1D).

### High RBD-IgG levels elicited by the first and second doses identified prior SARS-CoV-2 infection

**Figure 2:**
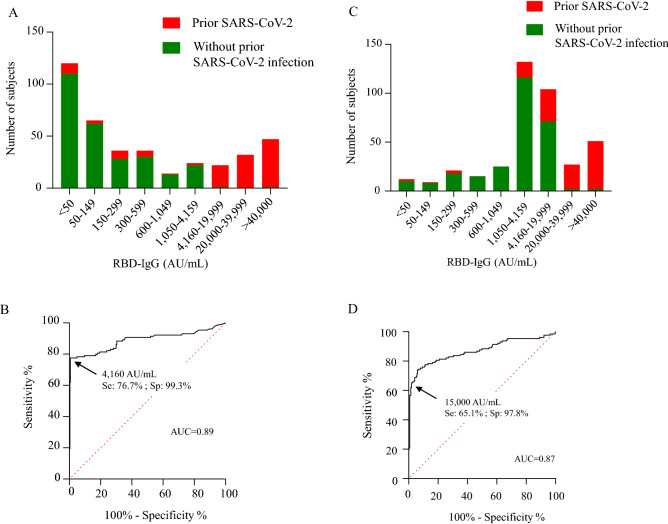
RBD-IgG levels among participants with or without prior SARS-CoV-2 infection. (**A**) Distribution of RBD-IgG levels in the entire population of participants 3 weeks after the first dose. (**B**) ROC evaluating the performance of RBD-IgG levels after the first dose, as a marker of prior SARS-CoV-2 infection; RBD-IgG > 4160 AU/mL: PPV: 98.0%; NPV: 89.8%; sensitivity: 76.7%; specificity 99.3%. (**D**) Distribution of RBD-IgG levels in the entire population of participants 6 weeks after the first dose. (**C**) ROC evaluating the performance of RBD-IgG levels after the second dose, as a marker of prior SARS-CoV-2 infection; RBD-IgG > 15,000 AU/mL: PPV: 93.3%; NPV: 85.3%; sensitivity: 65.1%; specificity: 97.8%.

A lack of RBD-IgG or low RBD-IgG concentrations were mainly seen in residents without prior SARS-CoV-2 infection, whereas high RBD-IgG levels were seen in residents with prior SARS-CoV-2 infection (Fig. 2A). After the first vaccine dose, a high RBD-IgG level was highly related to a prior SARS-CoV-2 infection (> 4160 AU/mL, PPV: 98.0%, NPV: 89.8%, sensitivity: 76.7%, specificity: 99.2%) (Fig. 2B).

After the second dose, a majority of residents had moderate/high levels of RBD-IgG (Fig. 2C). RBD-IgG values of over 15,000 AU/mL continued to be observed almost exclusively in residents with prior SARS-CoV-2 infection (PPV: 99.3, NPV: 85.3, sensitivity: 65.1%, specificity: 97.8%) (Fig. 2D).

### RBD-IgG vaccine response impacted by both interval since infection and nucleoprotein-IgG status

**Figure 3:**
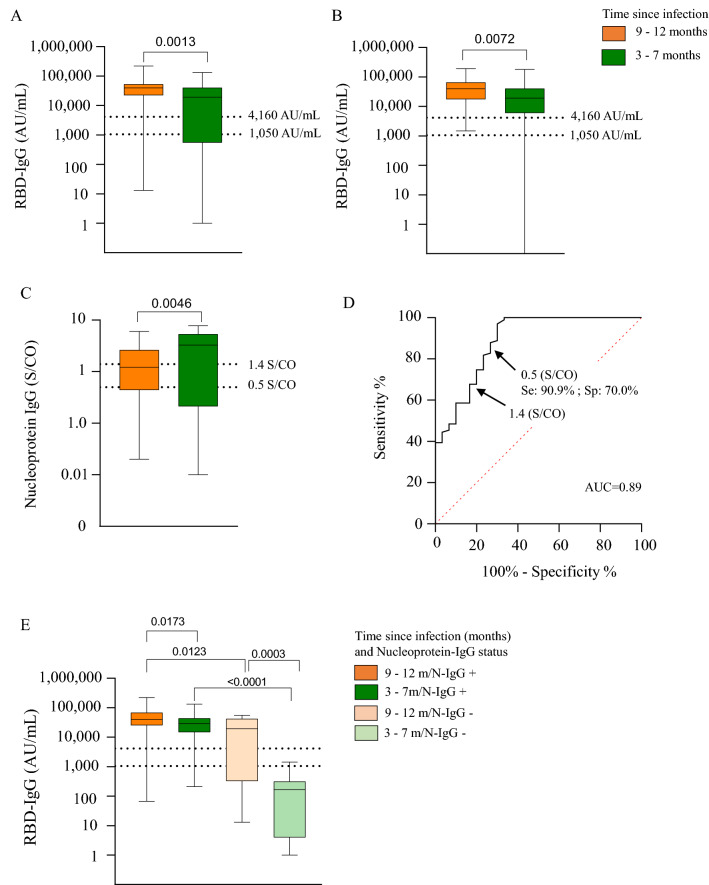
Impact of the interval between infection and vaccination and nucleoprotein-IgG status on RBD-IgG levels after the first dose. (**A**) RBD-IgG level according to the interval between SARS-CoV-2 infection and first dose. (**B**) RBD-IgG level according to the interval between SARS-CoV-2 infection and second dose. (**C**) Nucleoprotein-IgG S/CO value according to interval since SARS-CoV-2 infection. (**D**) ROC evaluating the performance of RBD-IgG S/CO index to predict a high RBD-IgG level after the first dose; RBD-IgG S/CO index > 0.5: PPV: 90.9%; NPV: 96.3%; sensitivity: 90.9% ; specificity: 70.0%). (**E**) RBD-IgG level after the first dose according to nucleoprotein-IgG status and interval since SARS-CoV-2 infection.

Residents infected during the first wave of the epidemic had a higher RBD-IgG response than those infected during the second wave (Fig. 3A,B). Conversely, the S/CO value of nucleoprotein-IgG was lower in residents infected during the first wave, compared to those infected during the second wave (Fig. 3C). Using the positive threshold retained for the study (S/CO: 0.5), detection of nucleoprotein-IgG was associated with a high RBD-IgG level after the first vaccine dose (PPV: 90.9%, NPV: 96.3%, sensitivity: 90.9%, specificity: 70.0%) (Fig. 3D). A lower sensitivity was observed when the threshold recommended by the manufacturer was used (S/CO: 1.4), (PPV: 92.5%, NPV: 51.0%, sensitivity: 75.5%, specificity 80.6%) (Fig. 3D). Among residents with prior SARS-CoV-2 infection, the increase in RBD-IgG levels between the first and second doses was obvious in residents who tested negative for nucleoprotein-IgG, but not significant in those who tested positive (Supplemental Fig. 2). Thus, the first vaccine dose generated the best antibody response in residents who had been infected during the first wave and who continued to maintain nucleoprotein-IgG at the time of that vaccination (Fig. 3E). Multivariate logistic regression analyses were done to identify the independent determinants of RBD-IgG levels over 4160 AU/mL. We observed an independent association between first wave SARS-CoV-2 infection, the presence of N-IgG for infections occurring during the second wave, and a good response to the first and second vaccine doses (Table 2).

**Table 2:** Univariate and multivariate binomial logistic regression analyses, describing the associations between NH resident characteristics and RBD-IgG response after 1st and 2nd vaccine dose. Significant values are in bold.

	Univariate			Multivariate		
	_C_OR	[95% IC]	p-value	_A_OR	[95% IC]	p-value
**High RBD-IgG after 1st dose (> 4160 AU/mL)**						
Age	1.02	[1–1.05]	0.099	–	–	–
Sex (female vs male)	1.26	[0.76–2.09]	0.366	–	–	–
			**< 0.0001**			< 0.0001
SARS-CoV-2 infection 9–12 months before vaccination	**211.36**	**[73.0–612.1]**	**< 0.0001**	**91.11**	**[22.3–372.1]**	**< 0.0001**
SARS-CoV-2 infection 3–7 months before vaccination	**86.72**	**[36.0–209.14]**	**< 0.0001**	**11.36**	**[3.29–39.2]**	**< 0.0001**
N-IgG (S/CO > 0.5)	**260**	**[104.4–647.4]**	**< 0.0001**	**102.6**	**[31.6–333.5]**	**< 0.0001**
**High RBD-IgG after 2nd dose (> 4160 AU/mL)**						
Age	1.00	[0.98; 1.02]	0.958	–	–	–
Sex (female vs male)	1.22	[0.79; 1.88]	0.363	–	–	–
			**< 0.0001**			**0.0024**
SARS-CoV-2 infection 9–12 months before vaccination	**22.26**	**[8.54; 58.1]**	**< 0.0001**	**6.05**	**[2.07–17.7]**	**0.0010**
SARS-CoV-2 infection 3–7 months before vaccination	**10.24**	**[5.3–19.8]**	**< 0.0001**	2.17	[0.91–5.18]	0.0823
N-IgG (S/CO > 0.5)	**36.21**	**[15.3–85.7]**	**< 0.0001**	**16.39**	**[5.97–45.0]**	**< 0.0001**

_*C*_*OR* crude OR. _*A*_*OR* adjusted OR.

### RBD-IgG level after natural immunization and its relationship to vaccine response

**Figure 4:**
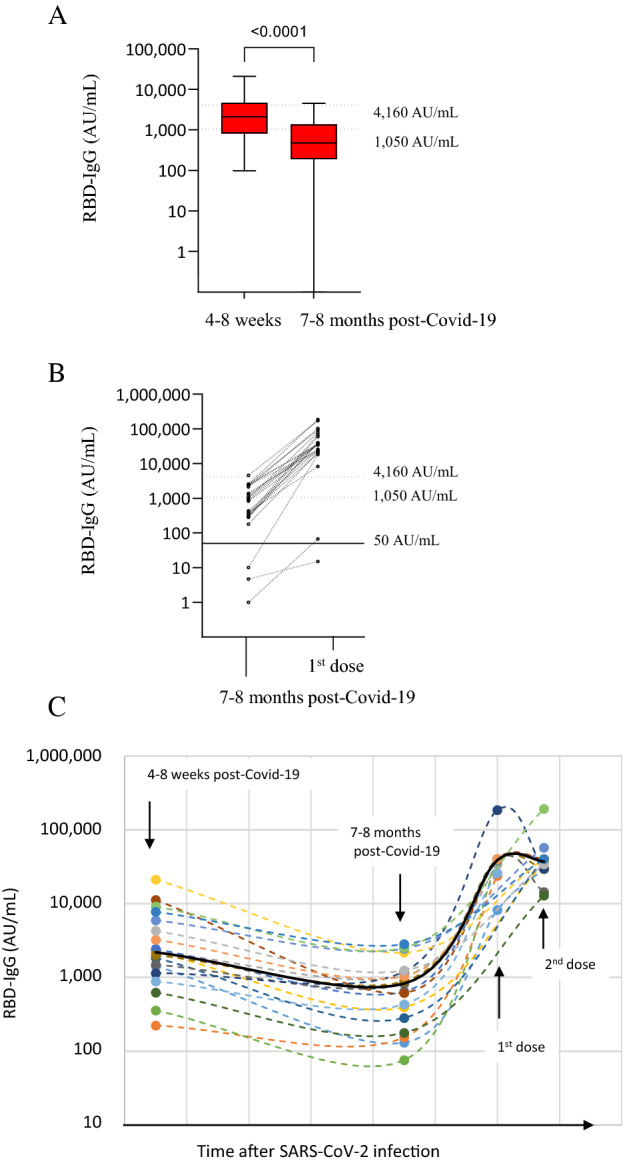
RBD-IgG levels after SARS-CoV-2 infection and relationship to vaccine response. (**A**) RBD-IgG level in response to natural immunization, 4–8 weeks (26 NH residents) and 7–8 months (n = 71 NH residents) after SARS-CoV-2 infection. (**B**) Individual changes in RBD-IgG levels after the first dose, in NH residents tested 7–8 months after SARS-CoV-2 infection. (**C**) Follow-up of RBD-IgG levels in NH residents tested after natural immunization and vaccination; black line indicates the mean RBD-IgG level.

RBD-IgG levels were quantified in convalescent sera collected in NH residents who had been infected during the first COVID-19 wave. RBD-IgG levels were moderate in NH residents tested 4–8 weeks after recovery, median (IQR): 2099 AU/mL (948–4018), and low in residents tested 6–7 months after recovery: 432 AU/mL (189–1193) (Fig. 4A). A median decay of 4.5 fold was observed in IgG-RBD levels of NH residents tested at the two time points. Among NH residents for whom pre-vaccine antibody results were available, we found that the humoral vaccine response was associated with RBD-IgG level induced by natural immunization (Fig. 4B). A weak correlation was observed between RBD-IgG levels after natural immunization and the first vaccine dose (R^2^ = 0.24, p = 0.03), (Supplemental Fig. 3A). A better correlation was observed between RBD-IgG levels 6–7 months after recovery and after the first vaccine dose (R^2^ = 0.68, p = 0.002), (Supplemental Fig. 3B). The follow-up of RBD-IgG levels in 17 NH residents tested after natural immunization and vaccination is presented in Fig. 4C.

## Discussion

Our study provides novel information on BNT162b2 vaccine response in older adults. Pre-vaccine SARS-CoV-2 infection, the interval passed since infection, and the persistence of a natural humoral immunity all influenced the antibody vaccine response. Assays that quantify anti-spike antibodies can be used on a large scale and involve a relatively low cost. Spike antibodies testing using automated assays constitutes a pragmatic approach to the assessment of vaccine response, and would constitute an advance towards a more tailored vaccine prophylaxis for NH residents.

It has been previously reported that RBD-IgG levels after a single vaccine dose in persons under 65 with a documented history of SARS-CoV-2 infection were at least as good as those obtained after two doses in persons without prior infection^24–26^. Our study, conducted in NH residents in whom the humoral response was carefully assessed after one and two BNT162b2 vaccine doses, shows similar results overall. This suggests that the first vaccine dose elicits a sharp recall response, such as that in immunocompetent younger adults. In those NH residents, most often high responders after the first dose, the second dose did not significantly increase RBD-IgG, suggesting that older adults with prior COVID-19 infection may not systematically need a second vaccine dose. Delayed administration of the 2nd dose may be preferable for recently infected NH residents, as previously suggested^27^. In the absence of a recommendation to assess immunity following vaccination, the provision of at least two vaccine doses remains nonetheless justifiable, given that the second dose is safe and well tolerated.

Older frail individuals respond weakly to vaccination due to immunosenescence, affecting their capacity to produce antibodies after suffering influenza or receiving the first dose of SARS-CoV-2 vaccine^25,28^. The elderly show a clearly reduced response to the COVID-19 vaccine^29^. In residents who had not suffered SARS-CoV-2 infection, we previously observed a poor humoral response or a lack of RBD-IgG subsequent to the first BNT162b2 dose^11^. After two doses, the RBD-IgG response remained lower overall than in younger participants^23,25^. It has been reported that in health care workers and adults, two doses of the BNT162b2 vaccine induce a response comparable to that observed after one dose in persons with prior SARS-CoV-2 infection^9,30–32^. Furthermore, a third dose of BNT162b2 substantially reduced the rate of severe illness in people over 60 years who were exposed to the SARS-CoV-2 Delta variant^33^. No threshold is currently recognized for protection against Covid-19. However, several studies have reported data associating anti-Spike antibody levels with protection against infection. Based on data from phase 2/3 studies, a threshold of RBD-IgG at 1880 AU/mL (264 BAU/mL) has been proposed for protection against symptomatic and asymptomatic SARS-CoV-2 Alpha variant infections^34^. This threshold was initially retained in France for persons identified as eligible for prophylaxis by monoclonal antibodies. Field data on health care workers^29^ and NH residents^23^ during the SARS-CoV-2 Alpha variant outbreak confirmed that such levels of RBD-IgG (1050 AU/mL and 993 AU/mL, respectively) correlate with COVID-19 protection.

Vaccine response is influenced by various host factors, which also result in a heterogeneous vaccine response such as age, immunosuppression and prior infection^7,35^. However, prevention strategies could nonetheless be improved by the identification of poor vaccine responders among residents at risk of severe forms of illness. As for immunocompromised individuals, those residents would need additional vaccine doses. The risk of not producing a high vaccine response after a single dose was significantly higher in residents with prior SARS-CoV-2 infection who tested negative for nucleocapsid-IgG. Absent or low RBD-IgG responses are more frequent in asymptomatic and mild forms of SARS-CoV-2 infection^36^. In addition, the clearance of circulating antibodies against SARS-CoV-2 depends on the level reached after recovery^37^. We previously observed that 5% of confirmed COVID-19 cases tested negative for nucleoprotein-IgG 6–8 weeks after recovery^17,18^. In this study, almost a quarter of the NH residents tested negative for nucleoprotein-IgG 3–12 months after recovery. Testing nucleoprotein-IgG could be a useful means of predicting RBD-IgG response after the first vaccine dose, and for estimating the possible additional benefit of a second dose after natural immunization. Our results show that a modified threshold for nucleoprotein-IgG, of 0.5 S/CO instead of the 1.4 S/CO threshold proposed by the manufacturer, had a stronger predictive value for a high humoral response to the first dose. Our results also found that the second factor associated with a higher RBD-IgG response was a longer delay between infection and vaccination, confirming previous results^38,39^. Strong anamnestic responses require an interval of at least 4 months between primary and secondary immune responses^40^. Prime and boost vaccination generate different plasma cells. Vaccine schedules aimed to induce long-lived plasma cells maintaining RBD-IgG levels at later time point. During the primary vaccination, short-lived plasma cells are generated with a rapid waning of antibodies, whereas the booster vaccination generates plasma cells providing long-lived immunity^41^. Spike antibodies are relatively easy to assess. The results of that assessment can be expressed in binding antibody unit/mL (BAU/mL), making comparisons of antibody levels easier. Despite such standardization, a definitive protective antibody level has yet to be determined. Hence, antibody testing is not currently recommended as a means of assessing immunity to SARS-CoV-2 following COVID-19 vaccination^42^.

This study has several strengths. Our study is based on a large number of participants, probably representative of the population of NH residents in France. Nursing homes followed guidance published by our Health Agency (in line with EuGMS guidance^17,43^), which explains that most NH residents with prior SARS-CoV-2 infection had PCR confirmation. Although NH residents have more comorbidities than most people of the same age, our results may also apply to frail older individuals living in the community. The main limitations of our study are the lack of a clinical outcome and the use of a unique mRNA vaccine (BNT162b2). Baseline antibodies prior to vaccination were only available for a small proportion of residents. Also, RBD-IgG levels were quantitated using an immunoassay, with concentration expressed in arbitrary units. We did not enumerate memory B cells and T cells that are critical for recall response against infection. In participants under 60 years of age who had recovered from SARS-CoV-2, the administration of a second vaccine dose close in time to the first showed no effect on the generation of memory B cells^44^. Neutralizing antibodies are considered a better marker of protection against severe form COVID-19, but such assays are not widely available in clinical practice. If neutralizing antibody levels appear to be a predictive marker of immune protection from severe COVID-19^37^, it remains to be demonstrated that RBD-IgG concentration is predictive of the level of protection against COVID-19.

Our study provides insight into the vaccination strategy involving the BNT162b2 vaccine. The need for a second dose 3 weeks after the first, in order to improve RBD-IgG response, is inconstant among NH residents with prior SARS-CoV-2 infection. NH residents who tested negative for RBD-IgG after the first vaccine dose are at high risk of producing a poor antibody response after two vaccine doses. For these participants, it remains to be determined whether a third dose may induce a significant RBD-IgG level. On the whole, these results suggest that consideration should be given to immunological monitoring in NH residents to optimize the vaccine response in this vulnerable population.

## Supplementary Information

Supplementary Figures.

## Data Availability

The datasets used and/or analysed during the current study available from the corresponding author on reasonable request.
