# Supplementary material for: Antibody response after first and second BNT162b2 vaccination to predict the need for subsequent injections in nursing home residents
Source: Sci Rep. 2022 Aug 12;12:13749. doi: 10.1038/s41598-022-18041-x (PMC9373891; doi:10.1038/s41598-022-18041-x)
Supplement: Supplementary file 1 — Supplementary Figures. [file 41598_2022_18041_MOESM1_ESM.pptx]

## Slide 1
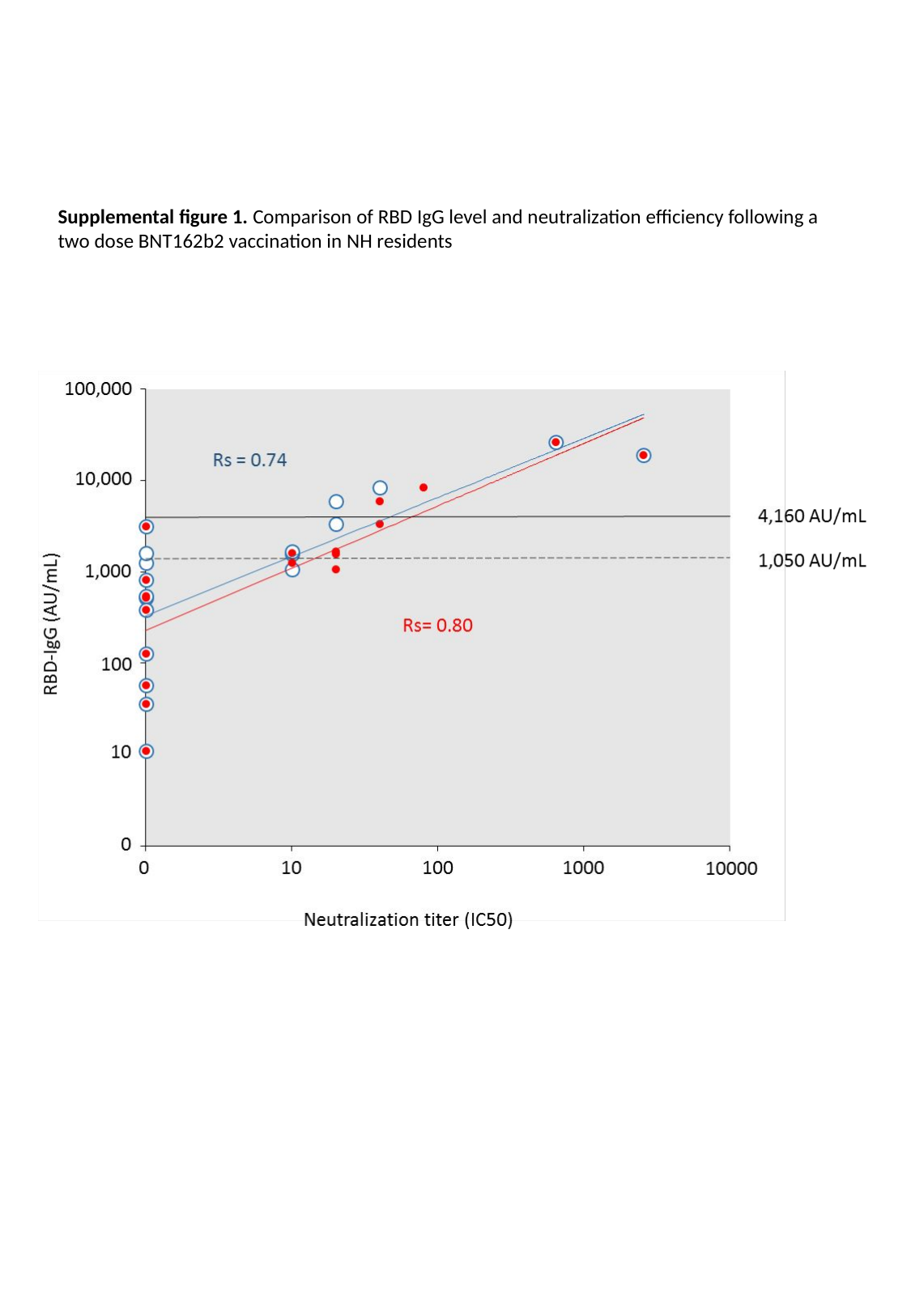

Supplemental figure 1. Comparison of RBD IgG level and neutralization efficiency following a two dose BNT162b2 vaccination in NH residents

## Slide 2
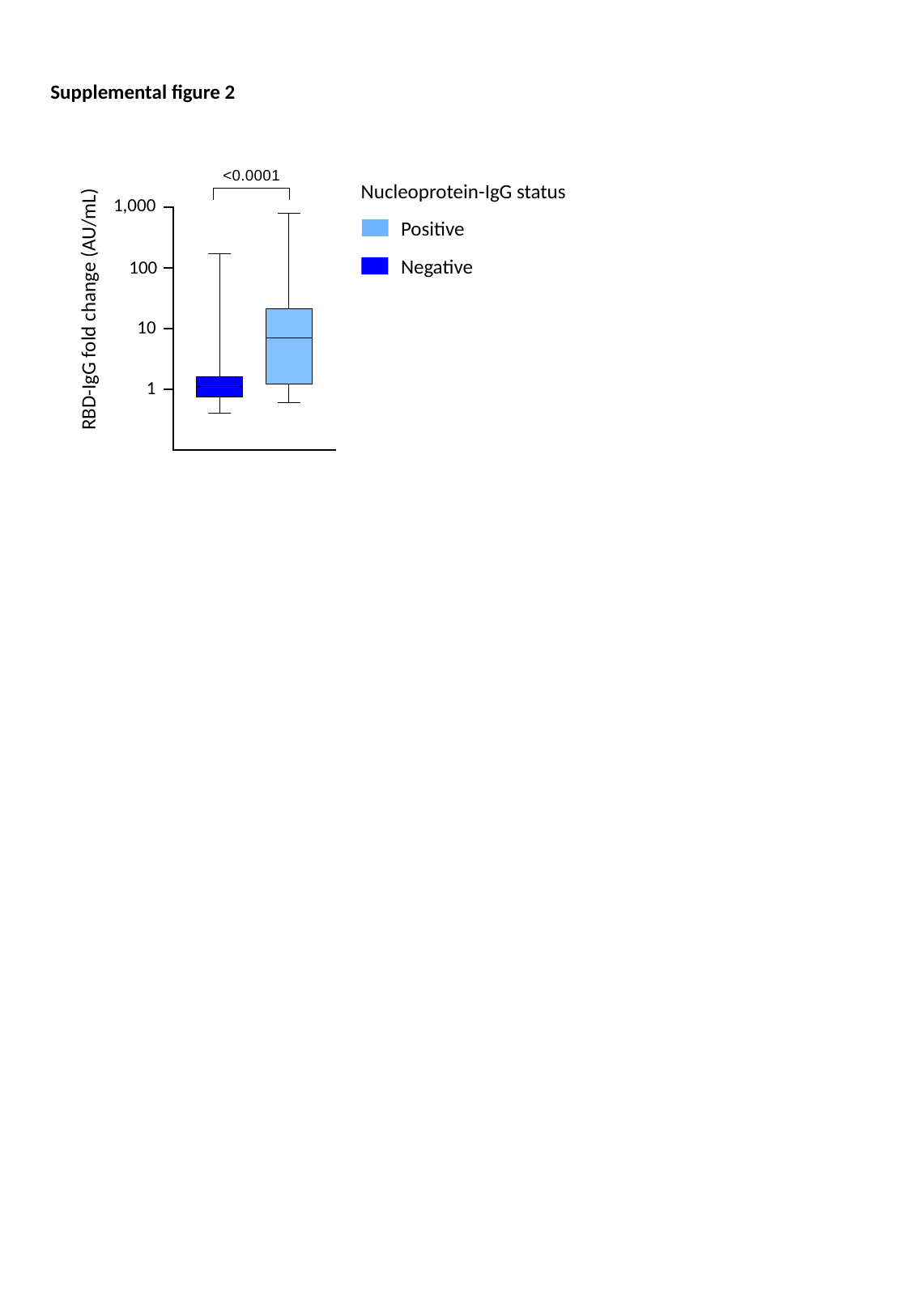

Supplemental figure 2
Nucleoprotein-IgG status
1,000
Positive
Negative
100
RBD-IgG fold change (AU/mL)
10
1

## Slide 3
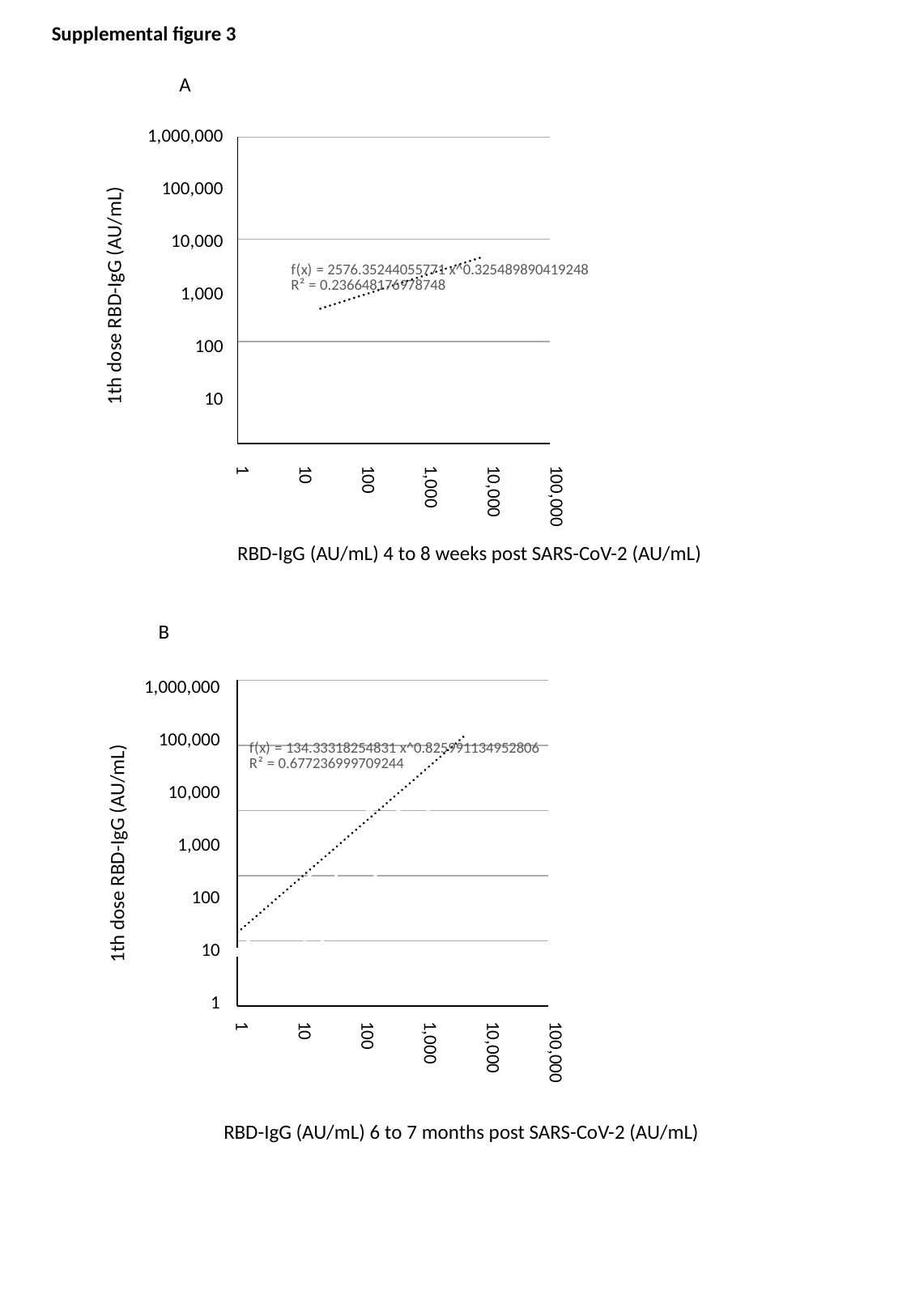

Supplemental figure 3
A
1,000,000
100,000
10,000
1,000
100
10
### Chart
| Category | Titrage IgG (protéine S) 1er dose |
|---|---|1th dose RBD-IgG (AU/mL)
100,000
10,000
1,000
100
10
1
RBD-IgG (AU/mL) 4 to 8 weeks post SARS-CoV-2 (AU/mL)
B
### Chart
| Category | Titrage IgG (protéine S) 1er dose |
|---|---|1,000,000
100,000
10,000
1,000
100
10
1
1th dose RBD-IgG (AU/mL)
100,000
10,000
1,000
100
10
1
RBD-IgG (AU/mL) 6 to 7 months post SARS-CoV-2 (AU/mL)
B

## Slide 4
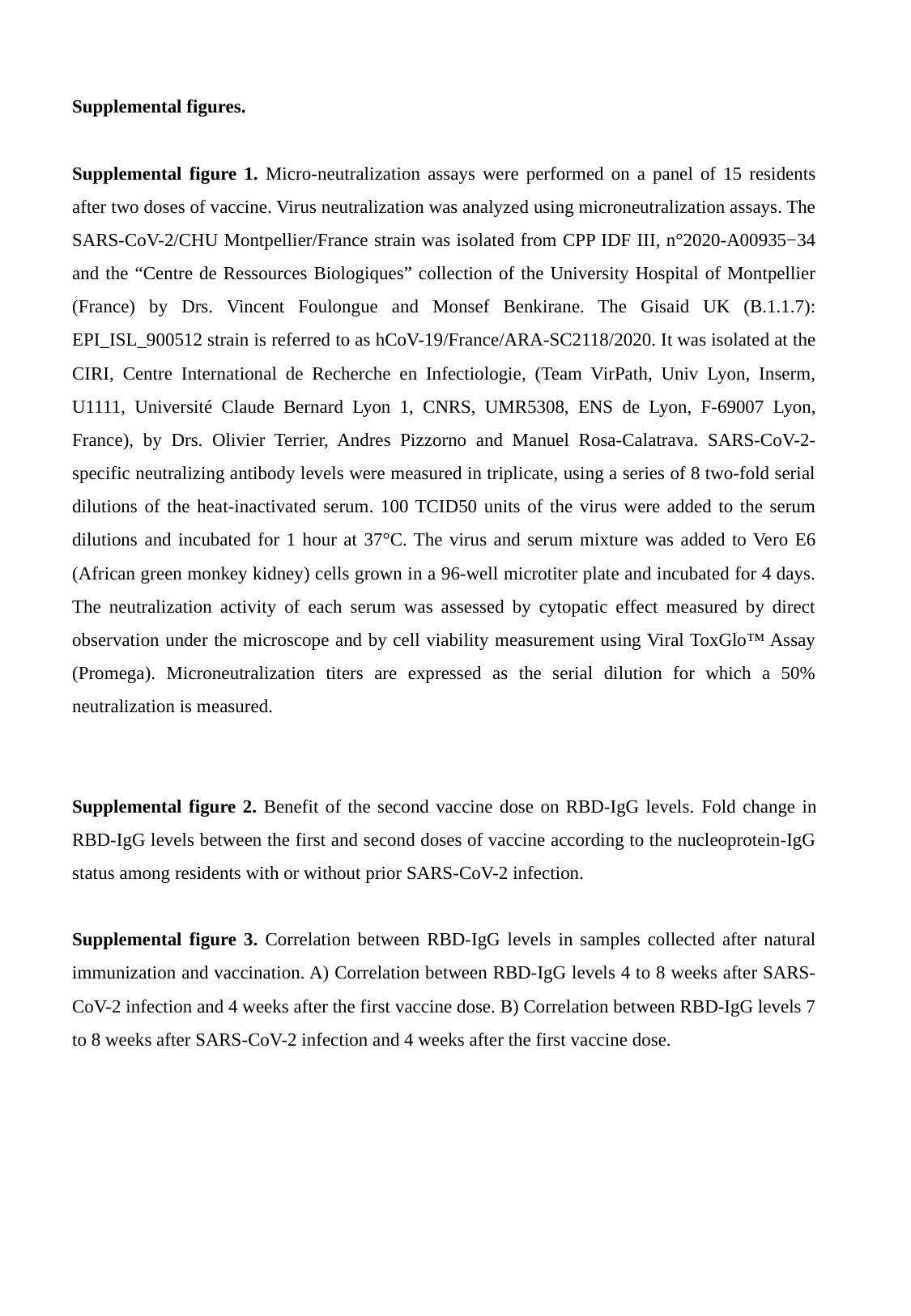

Supplemental figures.
Supplemental figure 1. Micro-neutralization assays were performed on a panel of 15 residents after two doses of vaccine. Virus neutralization was analyzed using microneutralization assays. The SARS-CoV-2/CHU Montpellier/France strain was isolated from CPP IDF III, n°2020-A00935−34 and the “Centre de Ressources Biologiques” collection of the University Hospital of Montpellier (France) by Drs. Vincent Foulongue and Monsef Benkirane. The Gisaid UK (B.1.1.7): EPI_ISL_900512 strain is referred to as hCoV-19/France/ARA-SC2118/2020. It was isolated at the CIRI, Centre International de Recherche en Infectiologie, (Team VirPath, Univ Lyon, Inserm, U1111, Université Claude Bernard Lyon 1, CNRS, UMR5308, ENS de Lyon, F-69007 Lyon, France), by Drs. Olivier Terrier, Andres Pizzorno and Manuel Rosa-Calatrava. SARS-CoV-2-specific neutralizing antibody levels were measured in triplicate, using a series of 8 two-fold serial dilutions of the heat-inactivated serum. 100 TCID50 units of the virus were added to the serum dilutions and incubated for 1 hour at 37°C. The virus and serum mixture was added to Vero E6 (African green monkey kidney) cells grown in a 96-well microtiter plate and incubated for 4 days. The neutralization activity of each serum was assessed by cytopatic effect measured by direct observation under the microscope and by cell viability measurement using Viral ToxGlo™ Assay (Promega). Microneutralization titers are expressed as the serial dilution for which a 50% neutralization is measured.
Supplemental figure 2. Benefit of the second vaccine dose on RBD-IgG levels. Fold change in RBD-IgG levels between the first and second doses of vaccine according to the nucleoprotein-IgG status among residents with or without prior SARS-CoV-2 infection.
Supplemental figure 3. Correlation between RBD-IgG levels in samples collected after natural immunization and vaccination. A) Correlation between RBD-IgG levels 4 to 8 weeks after SARS-CoV-2 infection and 4 weeks after the first vaccine dose. B) Correlation between RBD-IgG levels 7 to 8 weeks after SARS-CoV-2 infection and 4 weeks after the first vaccine dose.
